# Clinical Outcomes of Patients with Adrenal Incidentaloma - Hypertension being a Continuous Risk Factor for the Presence of Comorbidity: A Single Center’s Eight-year Experience

**DOI:** 10.2174/0115734056347340241009102228

**Published:** 2025-08-05

**Authors:** Gamze Akkus, Ulcaz Perihan Aksoydan, Fulya Odabaş, Hülya Binokay, Murat Sert, Tamer Tetiker

**Affiliations:** 1 Division of Endocrinology, Faculty of Medicine, Cukurova University, Adana, Turkey; 2 Internal Medicine Department, Faculty of Medicine, Cukurova University, Adana, Turkey; 3 Division of Biostatistics, Faculty of Medicine, Cukurova University, Adana, Turkey

**Keywords:** Adrenal incidentaloma, Hypertension, Risk factor, Comorbidity, Adrenal masses, Multivariable regression

## Abstract

**Background:**

Adrenal incidentalomas have increased over the past years. Although there are a lot of studies related to the frequency of adrenal masses and comorbidities, whether patients with functional or nonfunctional adrenal masses have higher risk is still a controversial issue.

**Methods:**

A total of 464 patients (female/male: 309/155) with adrenal incidentalomas were evaluated and followed up for 8 years. The patients were divided into 5 subgroups, including Autonomous Cortisol Secretion (ACS), Cushing Syndrome (CS), Pheochromocytoma (Pheo), Non-functional Adrenal Incidentalomas (NFAI), and Primary Aldosteronism (PA).

**Results:**

While 336 (72.4%) of the patients had NFAI, the others suffered from ACS (10.8%), CS (4.3%), Pheo (4.1%), and PA (8.4%), respectively. When comparing biochemical and demographical data, BMI (p=0.77), Hba1c (p=0.495), FPG (p=0.28), LDL (p=0.66), and HDL (p=0.521) were similar among the patients with functional and nonfunctional adrenal masses. The most common comorbidities were hypertension (n=259, 55.8%), diabetes mellitus (n=158, 34.1%), and dyslipidemia (33.4%), respectively. While 84 (32.4%) patients with hypertension had functional adrenal masses, the others (n=175, 67.6%) had non-functional adrenal incidentalomas. In subgroup analyses, hypertension was more common in patients with PA (87.2% *vs*. 72%, p=0.001) and ACS. In multivariable regression analyses, hypertension (p<0.001), cortisol (p=0.003), and aldosterone (p=0.04) levels were significantly correlated with functionality.

**Conclusion:**

Hypertension was the most common comorbidity in patients with adrenal adenomas, especially in functional adrenal adenomas related to serum cortisol and aldosterone levels.

## 1 INTRODUCTION

Adrenal Incidentaloma (AI) is an adrenal mass that can be found through imaging techniques applied for other reasons. As a clear definition, masses larger than 1cm in size are discovered on imaging for the evaluation of symptoms that are not suggestive of an adrenal problem. The prevalence of adrenal incidentaloma has increased due to the development of radiological imaging and estimated prevalence ranges from 4 to 10% in radiological studies [1, 2]. In addition, autopsy studies have also reported the prevalence of adrenal incidentaloma at a rate of 1.7-3.6% in patients above 50 years of age [3, 4]. Among autopsy and radiological series, the prevalence of AI increases with age, showing peak incidence in the fifth to seventh decades [5-7]. While gender differences have been related to the study populations in recent studies, female gender preponderance has been shown in many studies [8-12].

Most adrenal adenomas are benign cortical adenomas that may have secretory activity, although patients do not show clinical features of excess hormone findings [13, 14]. However, recent studies have highlighted that mild cortisol secretion could be seen in 20-30% of patients with AI [15, 16]. Subclinical Cushing syndrome or Autonomous Cortisol Secretion (ACS) can cause increased cardiovascular disease in patients with AI. Chronic mild exposure to the cortisol hormone is caused by the presence of metabolic abnormalities, including hypertension, insulin resistance, and hyperlipidemia [17]. All patients with adrenal incidentalomas should be evaluated in terms of functionality or nature of malignancy. There are many studies that have clarified the relationship between hormone-functional adenomas and comorbid diseases, including diabetes mellitus, hypertension, or dyslipidemia [18]. As we know, mild cortisol secretion or aldosterone excess adrenal incidentalomas are associated with frailty and increased cardiometabolic morbidities, such as hyperglycemia, hypertension, and dyslipidemia [19, 20]. However, we are still faced with some challenging questions, presented as follows: 1) is there any causative link between nonfunctional adrenal masses and comorbid diseases during the long-term follow-up? 2) If so, what is the difference between functional and non-functional adrenal masses?

We know that these studies include inherent bias in that diseased individuals are more likely to undergo imaging examinations than healthy ones and, therefore, the associations between adrenal incidentalomas and comorbid diseases may be explained by ascertainment bias. Therefore, we aimed to evaluate the frequency of functional adrenal incidentalomas in all patients with adrenal masses to better describe the presence of comorbid diseases in patients with functional or non-functional adrenal incidentalomas.

## 2 MATERIAL AND METHODS

### 2.1 Patients

We included patients who were referred to the division of endocrinology at the university hospital from 2015 to 2023. Patients were eligible for inclusion if they were aged 18 years or older and had an adrenal incidentaloma detected by cross-sectional imaging procedures. Retrospectively, the electronic medical files of all patients were reviewed manually. All specialist outpatient visits and hospital admissions have been coded with ICD-10 codes by the national database where they have been stored. We excluded those with any history of malignancy, major psychiatric disorders, or who were or still were on steroid treatment. All patients with AI underwent the following endocrine evaluation: baseline serum cortisol and Adrenocorticotropic Hormone (ACTH) at 8.00 a.m., followed by Dexamethasone Suppression Test (DST) measurement of cortisol levels, 24-hour urine-free cortisol and catecholamine levels, and plasma ratio aldosterone to renin activity.

### 2.2 Diagnostic Criteria

All patients with adrenal incidentaloma larger than 1 cm should be evaluated carefully, including clinical examinations and biochemical assessment. After the selection of the patients, they were divided into five different groups depending on the measurement of hormonal parameters according to the recent guidelines [18]; Non-functional Adrenal Incidentaloma (NFAI) was defined as adrenal masses larger than 1 cm (10 mm) without any hormone secretion. ACS was diagnosed when plasma cortisol levels were above 1.8 mcg/dL (50 mol/l) after the 1 mg dose and 2 days of 2 mg DST test, and there was a high degree of 24-hour urine cortisol levels, decreased ACTH (<10 pg/mL), and no Cushingoid features.

Patients with concomitant hypertension or unexplained hypokalemia were considered Primary Aldosteronism (PA); thus, increased Aldosterone (pg/mL) to Renin activity Ratio (ng/ml/h) (ARR>20) was considered as PA, and there were positive results obtained in the confirmatory tests (Saline infusion test).

Cushing Syndrome (CS) was diagnosed with the presence of Cushingoid features and normal or higher plasma cortisol levels after the 1 mg and 2 mg DST test, and increased levels of 24h urinary cortisol. In addition, at least one of the endocrine criteria needed to be met: 1) decreased ACTH levels (<10 pg/mL), 2) loss of diurnal cortisol rhythm (cortisol at midnight >7.5 mcg/dl), and 3) decreased ACTH levels response to CRH stimulation test.

Pheochromocytoma (Pheo) was diagnosed as increased (3-fold or higher) plasma-free or urinary fractioned metanephrine measurements of patients with AI.

We retrospectively collected data, such as age, sex, BMI (kg/m^2^), biochemical parameters, and concomitant diseases, including hypertension, diabetes mellitus, and dyslipidemia.

Hypertension (HT) was defined as systolic blood pressure of > 140 mmHg and diastolic blood pressure of > 90 mmHg under normal circumstances.

Diabetes Mellitus (DM) was defined as a Fasting Plasma Glucose (FPG) level of > 126 mg/dL and HbA1c (National Glycohemoglobin Standardization Program) > 6.5% or using an oral antidiabetic medication.

**Fig. (1):**
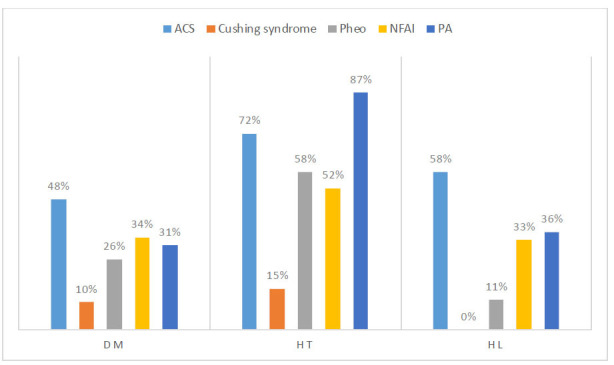
Comparement comorbidities of patients with adrenal incidentaloma. ACS: Autonomous Cortisol Secretion, Pheo: Pheochromocytoma, NFAI: Non-functional Adrenal Incidentaloma, PA: Primary Aldosteronism, DM: Diabetes Mellitus, HT: Hypertension, HL: Hyperlipidemia.

**Fig. (2):**
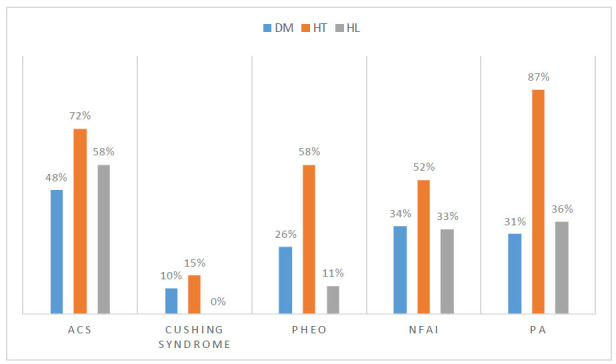
Comparement patients with adrenal incidentaloma according to comorbidites. ACS: Autonomous Cortisol Secretion, Pheo: Pheochromocytoma, NFAI: Non-functional Adrenal Incidentaloma, PA: Primary Aldosteronism, DM: Diabetes Mellitus, HT: Hypertension, HL: Hyperlipidemia.

Hyperlipidemia (HL) was diagnosed as a Low-density Lipoprotein (LDL) cholesterol level of >140 mg/dL, High-density Lipoprotein (HDL) level of < 40 mg/dL, Triglyceride (TG) level of > 150 mg/dL or using any antihyperlipidemic medication (Figs. **1** and **2**).

### 2.3 Surgical Treatments

If the patients had 1) clinically significant hormone excess; 2) radiologically suspicious features, such as increasing diameter of mass by at least 20% or the presence of heterogeneity; or 3) a mass larger than 6 cm, they were treated surgically.

According to these criteria, patients with functional adenomas (n=55, 43%) and non-functional adenomas (n=27, 8%) were treated with surgical therapy. Patients diagnosed with adrenal cortical neoplasia were reassessed according to Weiss criteria; thus, if 4 or more criteria were met, the mass was accepted as a carcinoma.

For PA, adenoma was not visualized by further diagnostic tests (imaging techniques) in the rest of the patients who were under medical treatment (mineralocorticoid antagonists).

### 2.4 Radiological Imaging

All abdominal Computed Tomography (CT) scans were evaluated by an experienced radiologist who reported the presence and number of adrenal tumors and their characteristics (largest size, margin, lateralization, *etc*.).

### 2.5 Assays

DHEAS (Dehydroepiandrosterone Sulphate), ACTH, and cortisol values were analyzed by using the enzymatic-labeled chemiluminescent immunometric assay method and chemiluminescence (Beckman DXI 800 auto-analyzer; Beckman Coulter Diagnostics, Fullerton, CA, USA), respectively. The High-performance Liquid Chromatography (HPLC) method was used to analyze urine cortisol and metanephrine values.

### 2.6 Statistical Analysis

The chi-square test was used to compare categorical variables between the groups. For the comparison of continuous variables between the two groups, the Student t-test or Mann-Whitney U test was used depending on whether the statistical hypotheses were fulfilled or not. Logistic regression analysis was performed to determine significant predictors of group (functional and non-functional) variables. Categorical variables have been expressed as numbers and percentages, whereas continuous variables have been summarized as mean and standard deviation and median and minimum-maximum where appropriate. All analyses were performed using the IBM SPSS Statistics Version 20.0 statistical software package. The statistical level of significance for all tests was considered to be 0.05 (SPSS reference: IBM Corp. Released 2011. IBM SPSS Statistics for Windows, Version 20.0 Armonk, NY: IBM Corp.).

## 3 RESULTS

### 3.1 Demographic Data of All Patients

464 patients were eligible and included in the current study. Female preponderance (155/309) was seen in all patients with adrenal masses. The mean age of all patients was 56.41±11.68 years (range 20-75).

### 3.2 Comparison of Functional and Non-functional Adrenal Masses

336 (72.4%) of the patients were diagnosed as having non-functional adrenal masses, whereas the others were diagnosed as having functional adrenal masses. The patients (54±12 *vs*. 57±12, p=0.018) with functional adrenal masses were younger than the patients with nonfunctional adrenal masses. When comparing biochemical and demographical data, BMI (p=0.77), Hba1c (p=0.495), FPG (p=0.28), LDL (p=0.66), and HDL (p=0.521) were found to be similar among the patients with functional and nonfunctional adrenal masses. Patients with functional adenomas had higher TG levels (170±86 *vs*. 156±90, p=0.04) than the patients with non-functional adenomas (Table **1**).

Among those with functional adenomas, 50 (10.8%) of the patients were diagnosed as ACS, 20 (4.3%) with CS, 19 with Pheo (4.1%), and 39 (8.4%) with PA. The mean age of all subgroups (ACS, CS, Pheo, PA, NFAI) at initial diagnosis was 51±9.6, 34±10.4, 39.4±13.4, 46.8±9.4, and 48.2±9.4, respectively. Hypertension was commonly seen in patients with PA and ACS compared to other patient groups (CS and Pheo).

Except for Pheo (female/male: 57.9%/42.1%), the female gender was more common in all subgroup patients (p=0.001). Mean cortisol measurements of the patients with ACS and CS were 12.09±4.6 and 20.77±7.5 (mcg/dL), respectively. Cortisol values after the 1 mg DST test of the patients with ACS and CS were 3.2±1.3 and 20.8±8.7 mcg/dL, respectively. The largest mass (mean diameter: 41.9±23.5) was seen in patients with Pheo; in addition, the sizes of masses in patients with ACS *vs*. CS were similar (30.7±11.4 *vs*. 36.3±2.3, p>0.05) (Table **2**). During a median follow-up of 8 years, none of the patients with ACS had developed overt Cushing syndrome.

### 3.3 Metabolic Parameters and Comorbid Condition

#### 3.3.1 Comorbidity of Diabetes Mellitus and Follow-up Period

Diabetes mellitus frequency in all patients was 34.05% (n=158). 115 of them (72.8%) had non-functional adrenal masses. In the comparison between the diabetic patients with functional adrenal masses and non-functional adrenal masses, the mean age (p=0.581), HbA1c (p=0.463), BMI (p=0.827), FPG (p=0.535), and TG (p=0.38) values were similar. In the case of hormonal parameters, mean cortisol (p=0.038), post-DST cortisol (p=0.00), urinary fractioned metanephrine (p=0.00), normetanephrine (p=0.02), and DHESO4 (p=0.04) parameters were significantly correlated with functionality (Table **3**).

12 (34.9%) of the diabetic patients with functional adrenal masses underwent surgical therapy with various indications. However, during the follow-up period, both of the two groups (surgical or non-surgical) had similar biochemical parameters, such as HbA1c (p=0.77), FPG (p=0.71), LDL (p=0.08), and TG (p=0.46).

#### 3.3.2 Comorbidity of Hypertension and Follow-up Period

The most frequent comorbidity in all patients was hypertension (55.8%, n=259) at the initial diagnosis. While 84 of them were diagnosed as functional masses (32.4%, n=84), the rest (n=175) were diagnosed as non-functional masses. Patients with functional masses were younger (57.7±9.7 *vs*. 60.7±9.3, p=0.016) compared to patients with non-functional masses. The other parameters, such as BMI (p=0.54), LDL (p=0.56), and TG (p=0.45) were similar among the patients with functional and non-functional masses. In the comparison of the hormonal parameters, the mean cortisol level (p=0.005), urinary fractioned metanephrine (p=0.03), and normeta- nephrine (p=0.02) were significantly correlated with functionality (Table **4**).

27 of the patients (32.1%) with functional masses were treated surgically (Pheo or PA) during the follow-up. The rest of the patients (67.9%, n=57) were followed with medical antihypertensive therapy.

The most commonly used antihypertensive medications were Angiotensin-converting Enzyme Inhibitors and Angiotensin Receptor Blockers (ACEI/ARB, 76.4%, n=198). The other antihypertensive medications were thiazide diuretics (57.9%), calcium channel blockers (53.3%), beta-receptor blockers (51.7%), spironolactone mineralocorticoid receptor blockers (13.5%), and alpha-blockers (13.9%), respectively.

#### 3.3.3 Comorbidity of Dyslipidemia and Follow-up Period

Dyslipidemia frequency was 33.4% (n=155) in all patients with adrenal masses. 45 of them (35.2%) were functional masses, and the others were followed up as nonfunctional masses. Mean LDL (p=0.66), HDL (p=0.52), and BMI (p=0.77) parameters were similar among the two groups. In the comparison of the subgroup analyses, 19 of the patients were operated for various indications.

In the multivariate analyses, hypertension (p<0.001), cortisol (p=0.003), and aldosterone (p=0.04) levels were significantly correlated with the presence of functionality (Table **5**).

## 4 DISCUSSION

The present study has demonstrated hypertension as the most common comorbidity in patients bearing adrenal adenomas, especially functional adrenal adenomas. With similar BMI, LDL, and TG parameters, the presence of HT was significantly correlated with functionality. We suggested that ACS and PA could be the strongest risk factors for the presence of comorbid diseases in patients with functional adrenal masses. This result could be related to CS having a lower risk for DM and HT in case of early diagnosis and treatment, but long-term mild cortisol secretion causes the presence of cardiometabolic diseases. The other most common comorbidities were diabetes mellitus and hyperlipidemia in all patients. In addition, our study was a long-term follow-up to investigate the natural history of functional (Cushing syndrome, autonomous cortisol secretion, primary aldos- teronism, and pheochromocytoma) and non-functional adrenal incidentalomas.

In our study, we demonstrated scientific knowledge, with female gender preponderance, to be more common in the ages of 40s and 50s. We also demonstrated untreated mild forms of hypercortisolism to have a deleterious effect on cardiometabolic profile.

Adrenal incidentalomas show different distribution in the population with regard to age, sex, size, and nature of the mass. Some studies have reported adrenal incidentalomas as a peak incidence in the fifth to seventh decades and as more frequent in females than males [21, 22]. However, some studies have argued that no gender differences exist [23, 24]. Terzolo *et al*. [25] showed the mean age of patients with adrenal incidentaloma to be 55 years and the female-to-male ratio to be 1.5. We demonstrated the mean age of all patients to be 56.36±11.7 and female to male ratio to be 1.9. We expected the mean age between the patients with functional (CS or Pheo) and nonfunctional adrenal incidentalomas to be different. Patients with functional adrenal incidentalomas, especially CS, were younger due to clinical features that allow early diagnosis. In addition, nonfunctional masses have benign radiological characteristics and absence of clinical and laboratory evidence of hormonal hypersecretion and are one of the most commonly seen subtypes of all AI [26]. Unlike ACS, the literature has limited data on metabolic disorders and their consequent cardiovascular outcomes in NFAI patients. Some studies have demonstrated a higher frequency of cardiometabolic conditions in these patients [27, 28]. A cohort study by Lopez *et al*. [29] showed the NFAI group to have approximately a 2-fold higher risk for diabetes than controls without adrenal tumors in the abdominal imaging. Cavalari *et al*. [30] reported similar findings in their study as they demonstrated a higher frequency of dyslipidemia, prediabetes, and hypertension, as well as waist circumference compared to the control subjects. The authors supposed that developing comorbid diseases are related to subtle, but not autonomous cortisol secretion. In the current study, the patients with non-functional adrenal incidentalomas (72.4%) were the main focus, being in line with the literature. A comparison of the demographical and biochemical parameters (BMI, FPG, LDL, TG) showed a similarity among the patients with functional or nonfunctional adrenal masses. Although hypertension was commonly seen in both groups, patients with functional adrenal adenoma had a higher risk of the presence of hypertension. The frequency of DM (34.2% *vs*. 33.6%) or dyslipidemia (35.2% *vs*. 32.7%) was similar in both groups with similar BMI, FPG, and Hba1c values. As we know, the prevalence of either type 2 diabetes or hypertension increases with age. It may be speculated that their association is an effect of aging or the lack of healthy control subjects could have affected the results of our study.

The frequency of comorbidities, including diabetes mellitus, hypertension, dyslipidemia, or osteoporosis, in the patients bearing adrenal adenomas was generally correlated with hormone excess features of the masses. More recent studies [31-33] have shown cortisol to be an independent risk factor for the presence of comorbidity in patients with adrenal adenomas. Metabolic alterations that augment cardiovascular risk in patients with autonomous cortisol secretion are known already and the association between this condition and adverse cardiovascular outcomes remains undefined. Di Dalmazi *et al*. [34] demonstrated that patients with non-secreting adenomas had a lower risk of DM and HT than patients with subclinical Cushing syndrome or autonomous cortisol secretion. In our study, hypertension was the most commonly seen comorbidity in patients with functional adrenal adenoma. Except for age, all patients with functional adenomas (ACS, CS, PA, Pheo) had similar biochemical parameters, but HT and type 2 diabetes were commonly seen in patients with PA and ACS. Initial fasting plasma glucose and LDL were similar between patients with ACS and CS and the existence of comorbidities, including HT, DM, and hyperlipidemia, was more common in patients with ACS. We suggest that maintained hypercortisolemia is a main contributing factor to these pathological alterations. In the multivariable regression analyses, hypertension and cortisol were also significantly correlated with functionality. We suggest that patients with ACS and PA have an increased risk of the presence of hypertension or type 2 diabetes.

The limitations of the current study can be attributed to the retrospective data analyses. This study was designed as a single-center study and some of the patient groups (PA, CS, Pheo) had a small sample size. In addition, we did not obtain results of healthy control subjects without adrenal masses to compare with patients with adrenal masses.

## CONCLUSION

In conclusion, this study has shown the association between the presence of HT in patients and adrenal adenomas, which may be a continuum risk independent of some important data, including BMI, FPG, Hba1c, and LDL values. Among the patients with functional adrenal adenomas, PA was still the main cause of secondary HT and maintained HT. ACS patients exhibited increased comorbidities, including DM, HT, and hyperlipidemia, as a result of continuous cortisol secretion. However, the risk was not equal in patients with CS. We assert this result to be related to early diagnosis and treatment of patients with CS.

## Figures and Tables

**Table 1:** Comparison of the biochemical and hormone paratemers between the patients’ with functional and non-functional adrenal masses.

N=464	Nonfunctional Mean±SD Median (min-max)	Functional Mean±SD Median (min-max)	p
Age (year)	57±12 59(20-89)	54±12 56(24-79)	**0.018**
BMI (kg/m^2^)	29.89±5.05 29.41(18.49-48.07)	29.62±5.99 29.33(17.63-48.41)	0.777
HBA1C (%)	6.3±1.5 5.9(4.0-16.2)	6.4±1.7 5.8(4.2-15.5)	0.495
FPG (mg/dL)	113±41 100(69-373)	113±47 97(69-404)	0.286
LDL (mg/dL)	130±40 128(36-274)	132±39 127(64-262)	0.660
HDL (mg/dL)	48±13 47(17-96)	49±12 48(22-87)	0.521
Triglyceride (mg/dL)	156±90 138(28-701)	170±86 144(64-555)	0.044
Renin activity ratio (ng/ml/h)	4.16±8.52 1.30(0.08-72.60)	3.33±6.83 1.00(0.15-50.00)	0.163
Aldosterone (pg/mL)	14.99±18.72 11.70(0.10-205.50)	30.72±57.37 15.95(0.10-420.30)	**0.002**
DHEAS04 (mcg/dL)	105.80±92.51 80.50(2.40-613.10)	73.66±68.76 44.50(4.70-274.90)	**<0.001**
Cortisol (mcg/dL)	10.91±4.45 10.00(3.89-33.40)	13.74±6.12 12.12(3.99-32.57)	**<0.001**
ACTH (pg/mL)	20.33±10.20 18.15(5.00-48.60)	17.35±11.29 15.60(2.00-47.10)	**0.001**
Cortisol post DST (mcg/dL)	1.07±0.55 0.97(0.24-6.03)	5.75±8.08 2.51(0.30-47.00)	**<0.001**
Hypertension (absent/present)	161 (47.9%)/175 (52.1%)	44(34.4%)/84(65.6%)	**0.009**
Diabetes Mellitus (absent/present)	221 (65.8%)/115 (34.2%)	85(66.4%)/43 (33.6%)	0.898

**Note: **Data are expressed as mean ± SD or as absolute value and percentage as appropriate. **Abbreviations: **FPG, Fasting Plasma Glucose; BMI, body mass index; LDL, Low-Density Lipoprotein; HDL, High-Density Lipoprotein, DHEA-S, dehydroepiandrosterone sulfate; ACTH, adrenocorticotropin hormone, HbA_1c_= glycosylated hemoglobin.

**Table 2:** Comparison demographical and biochemical parameters of the patients’ with functional masses.

N= 128	Autonomous Cortisol Secretion (n=50)	Cushing Syndrome (n=20)	Pheochromocytoma (n=19)	Primary Aldosteronism (n=39)	P^*^
Gender	-	-	-	-	**0.00**
Female	38 (76%)	20 (100%)	8 (42.1%)	22 (56.4%)	-
Male	12 (24%)	-	11 (57.9%)	17(43.6%)	-
Age (y)	51±9.6	34±10.4	39.4±13.4	48.2±9.4	**0.00**
BMI (kg/m^2^)	30.8±4.2	31.4±12.8	30.4±3.9	31.6±8.5	0.965
FPG(mg/dL)	119.5±50	99.5±20.5	116.2±33.2	110.2±29.2	0.479
HBa1c (%)	6.6±1.5	6.16±1.6	6.13±1.5	6.3±1.5	0.928
LDL (mg/dL)	136.7±43.6	141.7±24	139±29.2	131±39	0.596
Triglyceride (mg/dL)	165.4±77.4	167±53.9	158.07±90	172.8±80	0.47
Cortisol (mcg/dL)	12.09±4.06	20.7±7.56	13.2±6.43	12.3±4.0	**0.00**
ACTH (pg/ml)	12.3±7.8	5±12.3	30±5	30.0±5.0	**0.03**
Cortisol post DST (mcg/dL)	3.2±1.3	20.8±8.7	0.69±0.9	0.7±0.4	**0.00**
Maximum diameter (mm)	30.9±11.0	35.4±15.9	40.8±21.9	19.7±9.6	**0.00**
Comorbidity	-	-	-	-	-
Hypertension	72%(n=36)	15% (n=3)	57.9%(n=11)	87.2%(n=34)	**0.00**
Diabetes Mellitus	48% (n=24)	10% (n=2)	26.3%(n=5)	30.8%(n=12)	**0.037**
Hyperlipidemia	58% (n=29)	-	10.5% (n=2)	35.9% (n=14)	**0.00**

**Abbreviations: **FPG, Fasting Plasma Glucose; BMI, body mass index; LDL, Low-Density Lipoprotein; HDL, High-Density Lipoprotein; ACTH, adrenocorticotropin hormone, HbA_1c_= glycosylated hemoglobin.

**Table 3:** Comparison parameters of diabetic patients with functional and non-functional adrenal masses.

N=158	Non-functional	Functional	p
Female/Male	76/39	28/15	0.91
Age (y)	61.2 ±8.3	57.7±9.7	*0.581*
HBa1c (%)	7.38±1.84	7.71 ±2.12	*0.46*
FPG (mg/dL)	144.6±55.1	151.4 ±63.5	0.53
LDL (mg/dL)	123.06±41.4	122.7 ±36.3	0.825
BMI (kg/m^2^)	29.4±6.7	29.6±7.1	0.827
Triglyceride (mg/dL)	169.7±105.1	177.2±85.1	0.62
Cortisol (mcg/dL)	11.1±4.7	12.8±4.8	** 0.00**
ACTH (pg/ml)	19.5±9.2	16.9±9.3	0.07
Cortisol post DST (mcg/dL)	1.14±0.52	3.6±4.8	**0.00**

**Abbreviations: **FPG, Fasting Plasma Glucose; BMI, body mass index; LDL, Low-Density Lipoprotein; HDL, High-Density Lipoprotein, ACTH, adrenocorticotropin hormone, HbA_1c_= glycosylated hemoglobin.

**Table 4:** Comparison parameters of hypertensive patients with functional and non-functional adrenal masses.

*N=259*	*Non-functional *	*Functional *	*p*
Female/Male	114/61	54/30	0.89
Age (y)	60.7 ±9.3	57.7 ±9.7	**0.016**
Hba1c (%)	6.5 ±1.5	6.5 ±1.8	0.25
FPG (mg/dL)	122.5 ±44.1	120.7 ±52.7	0.38
BMI (kg/m^2^)	30.2 ±4.1	31.0 ±5.8	0.54
LDL (mg/dL)	126.8 ±40.6	130.0 ±41.8	0.56
Triglyceride (mg/dL)	151.4 ±82.9	179.2 ±84.5	0.45
Cortisol (mcg/dL)	10.8 ±4.4	12.6 ±4.6	** 0.005**
ACTH (pg/ml)	19.02 ±9.4	18.2 ±10.6	**0.04**
Cortisol post DST (mcg/dL)	1.16 ±0.6	3.1 ±3.8	**0.00**

**Abbreviations: **FPG, Fasting Plasma Glucose; BMI, body mass index; LDL, Low-Density Lipoprotein; HDL, High-Density Lipoprotein, DHEA-S, dehydroepiandrosterone sulfate; ACTH, adrenocorticotropin hormone, HbA_1c_= glycosylated hemoglobin.

**Table 5:** Risk factors of the patients’ with functional adrenal masses.

-	B	SE	OR	95%CI	P
Gender	-0.522	0.273	0.593	0.348-1.012	0.055
Hypertension	1.105	0.299	3.019	1.681-5.421	<0.001
Surgical treatment	2.087	0.351	8.063	4.056-16.027	<0.001
Aldesterone	0.010	0.005	1.010	1.001-1.020	0.046
Cortisol	0.081	0.027	1.084	1.028-1.143	0.003

## Data Availability

All data generated or analysed during this study are included in this published article.

## LIST OF ABBREVIATIONS

ACS: Autonomous Cortisol Secretion
CS: Cushing Syndrome
Pheo: Pheochromocytoma
NFAI: Non-functional Adrenal Incidentalomas
PA: Primary Aldosteronism
ACS: Autonomous Cortisol Secretion
ACTH: Adrenocorticotropic Hormone
DST: Dexamethasone Suppression Test
LDL: Low-density Lipoprotein
CT: Computed Tomography
HPLC: High-performance Liquid Chromatography

## STANDARDS OF REPORTING

STROBE and SAGER guidelines were followed.
